# Microglia and CNS Interleukin-1: Beyond Immunological Concepts

**DOI:** 10.3389/fneur.2018.00008

**Published:** 2018-01-23

**Authors:** Xiaoyu Liu, Ning Quan

**Affiliations:** ^1^College of Medicine, Institute for Behavioral Medicine Research, The Ohio State University, Columbus, OH, United States; ^2^Division of Biosciences, College of Dentistry, The Ohio State University, Columbus, OH, United States

**Keywords:** cytokine, neuroinflammation, brain, neuromodulation, IL-1R1

## Abstract

Activation of microglia and expression of the inflammatory cytokine interleukin-1 (IL-1) in the CNS have become almost synonymous with neuroinflammation. In numerous studies, increased CNS IL-1 expression and altered microglial morphology have been used as hallmarks of CNS inflammation. A central concept of how CNS IL-1 and microglia influence functions of the nervous system was derived from the notion initially generated in the peripheral immune system: IL-1 stimulates monocyte/macrophage (the peripheral counterparts of microglia) to amplify inflammation. It is increasingly clear, however, CNS IL-1 acts on other targets in the CNS and microglia participates in many neural functions that are not related to immunological activities. Further, CNS exhibits immunological privilege (although not as absolute as previously thought), rendering amplification of inflammation within CNS under stringent control. This review will analyze current literature to evaluate the contribution of immunological and non-immunological aspects of microglia/IL-1 interaction in the CNS to gain insights for how these aspects might affect health and disease in the nervous tissue.

## Introduction

Changes in microglial morphology are one of the most common findings of neuropathology in almost all CNS diseases. Long regarded as the resident immune cells in the immunologically temperate environment of the CNS, the resting spider-shaped microglia become deramified and amoeboid in activated states (1). This shape shift has been observed in acute brain injury (2), various neurodegenerative diseases such as Alzheimer’s disease and Parkinson’s disease (3), CNS autoimmune diseases such as multiple sclerosis (MS) (4, 5), convulsive disorders such as epilepsy (6), and even affective disorders including major depression (7, 8), anxiety disorders (9), and autism (10). These evidences led to the hypothesis that microglial activation is a significant common cause of neuropathology in these diseases (11), although microglial morphological changes alone may not always reflect the precise activation status (12, 13) among the variegated states that these cells can adapt.

Another salient-related observation in CNS diseases is the increased expression of the inflammatory cytokine interleukin-1 (IL-1). IL-1 is a master regulator of inflammatory reactions in the immune system, capable of activating innate immunity by inducing the expression of numerous inflammatory cytokines and chemokines, eliciting leukocyte infiltration into the inflammatory loci, increasing phagocytic and bactericidal activity of immune cells, enhancing activity of the complement system, and facilitating the activation of the adaptive immune responses (14). Correlations of plasma or CNS levels of IL-1 and disease severities in the abovementioned CNS diseases have been widely reported (15–20), although there are also many reports that fail to show correlation between plasma IL-1 level and the presence of disease symptoms in these diseases (21, 22).

Combining increased IL-1 expression and microglial activation as a composite indicator of pathogenesis in CNS diseases seems to be an attractive idea because it might overcome the shortcomings of using each of them separately. In peripheral tissues, increased IL-1 expression is tightly linked with macrophage activation during inflammation (23); in the CNS, neuroinflammation may not display the entire panoply of peripheral inflammation, e.g., swelling may not occur during neuroinflammation, but increased expression of IL-1 by brain tissue together with morphological changes in microglia appear to be a frequently observed phenomenon in both human neuropathology and animal models of brain diseases (16, 24–28). Adding increased brain IL-1 expression to supplement microglial morphology changes would further specify that the changes in microglia are part of the inflammatory microgliosis.

The villainization of microglial activation and CNS IL-1 expression, however, has been countered by the teleological argument: is the CNS designed to have microglial activation and IL-1 expression just to cause pathology (29)? Such incredulity has been substantiated by the facts that blocking inflammatory microglial activation can lead to the exacerbation of some symptoms of certain CNS diseases (30–34) and clinical benefits of drug treatments for reducing microglial activation or IL-1 activity have been demonstrated recently in stroke patients (35), but the utility of this strategy for the treatment of the vast majority of the above-mentioned diseases remains to be firmly established after it has been advocated for at least 10 years.

Besides their pathogenic roles, functions of microglial activation and IL-1 expression in CNS development, repair, and physiological activity have been intensely studied recently. This endeavor has yielded tremendous advances, revealing many new areas of understanding on the non-immunological functions of IL-1 and microglia in the CNS (11, 36–39). These new findings in the realm of the positive contributions of microglia and IL-1 in the CNS educe the critical inquiry: how would the immunological and the non-immunological aspects of IL-1 and microglial functions coordinate or disrupt each other to affect health and disease?

## The Promises and Limitations of the Inflammatory Paradigm

Although current literature is beginning to shed light on the multifaceted roles played by microglia and CNS IL-1, the simple inflammatory paradigm, *viz*., increased CNS IL-1 expression together with microglial activation amplifies neuroinflammation and causes neuropathology, has accrued formidable experimental support. The following rationales have propelled the research in this area: (1) inflammatory process is designed to sequester and kill infectious pathogens and contain necrotic tissue damage; this entails the induction of proinflammatory cytokines and chemokines, recruitment of leukocytes, and the production of bactericidal reactive oxygen species (ROS), all potentially neurotoxic, (2) CNS is immunologically privileged site, bystander neuronal casualty from inflammation is likely to cause irreversible damage to this delicate tissue which lacks significant regenerative potential and expandable volume, and (3) neuroinflammation could lead to CNS autoimmunity resulting in attacks by immune cells to CNS antigens which are normally dormant.

The induction of CNS expression of proinflammatory cytokines including IL-1 has been shown in animal models of acute brain injury (40–43), Alzheimer’s disease (44, 45), Parkinson’s disease (25), CNS autoimmunity (46), anxiety disorder (47–50), major depression (51–53), and autism (54). *In vitro* studies were the first to show that inflammatory cytokines, especially IL-1 and TNF-α, can cause neuronal death by the direct effects of these cytokines on neurons or indirectly by glial production of neurotoxic substances (55–58). Similarly, a few chemokines have also been found to possess neurotoxic activity. CXCL4 (59), was the first to be identified in this regard; more recent studies also found CCL11 (60), CXCL2 (61) can exert neurotoxic effects on cultured neurons.

Neurotoxicity from infiltration of peripheral leukocytes has also been documented. Typically, in experimental conditions that resulted in leukocyte infiltration into the brain, the infiltrated peripheral myeloid cells show higher expression levels of proinflammatory cytokines than resident glial cells (62–64). Thus, entrance of peripheral leukocytes into the CNS may represent a more severe type of CNS inflammation. Reduction of leukocyte infiltration by blocking vascular adhesion molecules or chemokine activity has been shown to improve outcomes in acute brain injury (65, 66) and CNS autoimmune diseases (67, 68). Interestingly, although infiltration of peripheral leukocytes into the CNS is generally not a common observation in human affective disorders, this phenomenon occurs in several animal models of stress- or inflammation-induced depression and/or anxiety (69, 70). Preventing CNS infiltration of IL-1 expressing leukocytes protected animals from displaying depressive and/or anxiety-like behaviors in these models (64, 71).

Other studies demonstrated a pathogenic role of oxidative stress. Blocking inflammation-induced production of ROS or ROS activity alleviates neural damage in cerebral ischemia (72–74) and cerebral hemorrhage (75), reduces depressive and anxiety-like behaviors caused by peripheral inflammatory stimulation (76), lessens certain symptoms induced in an Alzheimer’s mouse model (77). In addition, ROS production and antioxidant defense imbalance has been observed in acute brain injury (78, 79), inflammation-induced depression and anxiety, and neurodegenerative diseases (80, 81). These evidences support the hypothesis that oxidant/antioxidant imbalance downstream of IL-1-stimulated microglial activation is a common feature for both acute and chronic neuropathology and their attendant psychopathology (82, 83).

The possibility of bystander damage of CNS inflammation is best demonstrated in situations of CNS infection. Initially, post-infectious neurological dysfunction was thought as a consequence of permanent damage caused by the invading pathogens and the specific immune responses to the pathogen (84). However, patients who survived CNS infection sometimes show deficits implicating brain regions beyond the foci of the initial infection (85) and animal studies show chronic neuroinflammation may persist after the acute infectious pathogens have been eradicated (86). Thus, off-target inflammatory activity may contribute to post-infectious neuropathology.

Further bolstering the case for malignant inflammatory effects are the findings that endogenous CNS antigens that normally do not induce autoimmune attacks can be turned susceptible when CNS inflammation is present. In experimental autoimmune encephalitis (EAE), the brain endothelial receptor for IL-1 (IL-1R1) and infiltration of myeloid cells expressing IL-1β was found to be required for the induction of illness (63). Because IL-1β-expressing myeloid cells are involved in inflammatory activity, not antigen specific immunity, these results point to the importance of inflammation in facilitating autoimmune activity of the CNS. Dysregulation of microglia may also contribute to the pathogenesis of PANDAS (Pediatric Autoimmune Neuropsychiatric Disorders Associated with Streptococcal Infections) which was thought to be caused by the induction of post-infectious cross-reactive autoantibodies against CNS tissue (87–89). Therefore, neuroinflammation might augment autoimmune activity-related neuropathology.

A major recent advance in the field of inflammation is the discovery of inflammasomes. Inflammasomes are protein complexes that act as intracellular sensors for the disruption of homeostasis (90). They include NOD like receptors and ASC (apoptosis-associated speck-like protein containing a caspase recruitment domain). Inflammasomes regulate IL-1 and IL-18 activity by regulating caspase-1, which cleaves inactive pro-IL-1 and pro-IL-18 to derive the active IL-1 and IL-18. This intermediate step allows preformed pro-IL-1 and pro-IL-18 to be quickly activated, ensures inflammation occurs through the priming stage (the synthesis of pro-IL-1 and molecules of the Inflammasomes) and the activation stage (the generation of mature inflammatory cytokines), thus providing a mechanism that requires “two-hit” to induce inflammation, allowing finer control of the timing and the magnitude of inflammatory cascade. In addition, inflammasomes are sensitive to stimulations by internal disturbance, such as misfolded or aggregated proteins and aberrant products of energy metabolism, broadening the range of inflammation inducers beyond infectious stimuli (90). In ischemic or hemorrhagic stroke models, expression of the NLRP3, a known microglial inflammasome (91, 92) component, was increased, and specific blockade of NLRP3 reduced stroke induced neural damage and functional deficits (93, 94). Several NLRP3 component proteins were also induced in the pathological tissues in Alzheimer’s disease (95). Aggregated or fibrillary α-synuclein, a known pathogenic factor for Parkinson’s disease also stimulates the activation of NLRP3 (96). Activation of NLRP3 has been documented in depression and anxiety and both pharmacological blockade of NLRP3 or gene deletion of NLRP3 reduces depressive behavior and anxiety in animal models of these disorders (97, 98).

The inflammatory paradigm, increased brain IL-1 expression and microglial activation drives the progression of CNS diseases, has gained further momentum from studies that used drugs to inhibit IL-1 activity and/or microglial activation. A naturally occurring antagonist for IL-1 is the IL-1 receptor antagonist (IL-1ra). A recent meta-analysis shows treatment with IL-1ra reduces infarct volume by 36% in animal models of cerebral stroke with more reliable efficacy if the drug is delivered into the cerebral ventricle than into the blood (99). IL-1ra was also effective in blocking stress-induced depression and anxiety (51, 52, 100), and in improving clinical outcomes in experimental epilepsy (101, 102). Minocycline, a tetracycline derived antibiotics, has been found to inhibit inflammatory microglial activation (103). Specifically, activated microglia could differentiate into multiple activated states: the most inflammatory type is designated as M1 and the most anti-inflammatory type is designated as M2. Besides changes in morphology, M1 microglia express inflammatory cytokines including IL-1, TNF-α, and iNOS, whereas the M2 microglia express TGF-β, IL-4 or IL-10, and arginase 1. Treatment with minocycline selectively inhibits M1 microglial activation (104). Pretreatment with minocycline provides neuroprotection against excitotoxicity (105), oxidative stress (106), reduces symptoms in animal models of Parkinson’s disease (107), cerebral stroke (108), epilepsy (109), and stress-induced depression (110). In EAE, a model of MS, minocycline treatment was found to be effective in reducing disease severity and histological outcomes when used in combination with other conventional treatments (111–116) or alone (117, 118). The promise of using drugs against IL-1 and microglial activation to treat CNS diseases is attested by the current clinical trials that use IL-1ra to treat cerebral stroke (119–121), fatigue in Sojegren’s syndrome (122), and minocycline to treat cerebral stroke (123, 124), cerebral hemorrhage (125), Parkinson’s disease (126, 127), epilepsy (128), bipolar and treatment resistant depression (129, 130), and schizophrenia (131). These trials have generated promising results, although large scale clinical tests are still needed. In human MS trials, minocycline treatment reduced MS lesion detected by MRI (132) and reduced the risk of conversion of patients with first demyelinating event from progressing to MS (133).

The notion that all CNS diseases can be effectively treated by inhibition of IL-1 driven microglial activation, of course, is overly simplistic. A dramatic cautionary tale is supplied by a study that investigated the role of IL-1β in Alzheimer’s disease. Beta-amyloid aggregation in this disease causes the formation of senile plaques. Transgenic overexpression of IL-1β unexpectedly reduced plaque formation, despite inducing robust neuroinflammation (134). In another surprising study, chronic unpredictable stress induced depressive-like behavior; stimulating rather than inhibiting microglia provided anti-depressant effects (135). These results highlight the limitation of the inflammatory paradigm and suggest non-immunological functions of IL-1 and microglia should be examined.

## IL-1 and Microglia as Neuromodulators

The neurophysiological functions of IL-1 were first investigated in temperature-sensitive neurons because IL-1 was identified as the endogenous pyrogen that mediates fever after bacterial infection. In the temperature control center of the brain, the preoptic area of the hypothalamus, IL-1 decreased the sensitivity of warm-sensitive neurons, but increased the sensitivity of cold-sensitive neurons, thereby modulating the thermoregulatory circuits in a manner consistent with its pyrogenic role (136, 137). This IL-1 activity is not related to neuroinflammation but could be an indirect effect because it can be blocked by inhibitors of cyclooxygenase, which catalyzes prostaglandin production downstream of IL-1 signaling (138). IL-1 may even mediate neurophysiological effects under sterile condition. A good example here is its role in regulating normal sleep. IL-1 is expressed in the brain with a diurnal rhythm, and increased expression of IL-1 is associated with increased spontaneous sleep whereas inhibition of IL-1 activity reduces sleep (139). Interestingly, neuronal IL-1 expression and indirect activation of neurons by CNS IL-1 may underlie the sleep promoting effects of IL-1 as it can promote synchronization of sensory neurons (140). Other indirect electrophysiological effects of IL-1 have been reported in neurons of the supraoptic (138) and paraventricular nucleus of the hypothalamus (141). Direct effects of IL-1 on neuronal excitability have also been reported (138); but the mechanism for this function remains unclear. IL-1 was found to inhibit Ca+ channel currents (142), reduce GABA A receptor-mediated response (143), inhibit NMDA receptor-mediated synaptic transmission (144), activate non-selective cationic conductance (145), potentiate voltage-dependent sodium currents in nociceptive neurons (146, 147), and increase voltage-gated potassium currents (148), depending on the different types of neurons studied. In the dentate gyrus, IL-1 may facilitate or inhibit the generation of long-term potentiation (LTP) (149, 150), a critical neural mechanism for learning and memory. LTP occurs as persistent increases of synaptic strength after high-frequency synaptic stimulation, thus potentially coding for learning or memory processes. Interestingly, the learning process itself causes hippocampal expression of non-inflammatory levels of IL-1, which in term, helps maintain LTP (150, 151). These scattered reports of IL-1-mediated neurophysiological effect appear incongruent at first glance, but an emerging theme is IL-1 can modulate sensory system of the nervous system in order to modulate perception and learning. It should be noted that such modulation may have time-dependent and concentration-dependent variable effects. Acute IL-1 effects may heighten perception and learning whereas chronic IL-1 effects may reduce sensory function, retard learning, and cause fatigue (147, 148, 152–154). Similarly, low levels of IL-1 may facilitate memory whereas high levels of IL-1 or complete blockade of IL-1 signaling may impair memory (155). One difficulty in the past is to identify IL-1 receptor expressing neurons and the observed neuromodulatory effects of IL-1 may be attributed to the indirect action of IL-1 that might elicit neural active substances such as nitric oxide (156), ATP (157), or prostaglandins (145). Recently, we have developed a knockin mouse line that allowed the tracking of IL-1 receptor expression cells in a cell type specific manner. We now have unpublished results that show IL-1 type 1 receptor is preferentially expressed in numerous sensory brain regions.

Another neuromodulatory role of IL-1 is on neurogenesis. Reduced production of new neurons in adult hippocampus has been linked with the pathogenesis of depression (51). This role of IL-1 was initially observed in animal models of interferon-γ (IFN-γ) treatment. IFN-γ is used to treat hepatitis C but has the unfortunate side effect of causing depression. In a rat model of IFN-γ-induced depression, hippocampal IL-1β expression and reduced neurogenesis in dentate gyrus was induced and administration of IL-1ra blocked these effects together with the depressive behavior (158). This mechanism is also operative in chronic stress induced depression: chronic mild stress was found to induce IL-1β expression in the hippocampus, reduce neurogenesis, and cause depressive like behavior in wild-type mice. These changes were absent in IL-1 receptor knockout mice or transgenic mice that express IL-1ra in the brain (159). That IL-1 driven microglial activation may be involved in this phenomenon is further supported by the evidence that inhibition of NFκB activation blocked the antineurogenic and depressive effects of the stress (160). Brain IL-1 is known to induce microglial NFκB activation (161). It should be noted that chronic mild stress dose not induce leukocyte infiltration into the brain; thus this IL-1-mediated microglial activation may not represent an immunological neuroinflammation. In addition, the antineurogenic effect of IL-1 may also be concentration dependent as IL-1 can facilitate neuronal survival by promoting the expression of nerve growth factors (NGFs) (162).

Induction of neurotrophic factors is one of the early observations on IL-1-mediated non-immunological neural effects (163, 164). In traumatic brain injury, increased NGF expression follows the increased expression of IL-1 in the wounded tissue. Injection of IL-1ra blocked NGF and the associate neuroreparative responses (165). IL-1 has also been found to stimulate neurotrophin-3 and brain derived neurotrophic factor, supporting neuronal survival and neurite growth (166, 167). However, interaction between IL-1 and the neurotrophic factors can also be a double-edged sword. Systemic IL-1, not central IL-1, have been reported to reduce hippocampal BDNF expression (168); while acute intracerebral IL-1 caused the expression of neurotrophic factors and neuroprotection, subacute IL-1 (4 days of IL-1 injection) caused the opposite effects (169); IL-1 can also increase neuronal vulnerability by increasing the surface expression of the p75 neurotrophin receptor (170).

Physiological activities of IL-1 in the brain also include neuroendocrine functions. Psychological and metabolic stress induced ACTH and glucocorticoid responses were reduced in IL-1 receptor knockouts or transgenic mice overexpressing brain IL-1ra (171). Intracerebral administration of IL-1 is known to induce CRH release (172) and psychological stress has been shown to induce brain IL-1 expression (173). Therefore, brain IL-1 could mediate physiological response to stress by stimulating the production of the immunosuppressive hormone glucocorticoid. In addition, IL-1 acting in the brain can stimulate brain metabolism despite hypoglycemia. Neuronal IL-1 synthesis was found to be induced by stimulation of AMPA receptors on neurons and the resulting release of IL-1 can stimulate glucose uptake by neurons in an autocrine or paracrine fashion (174). It is interesting to speculate that these physiological activities of IL-1 might coordinate with the immune activities of IL-1 such that hyper-inflammation may be prevented and brain energy usage may be spared even when immune activity might be energetically costly. It is interesting to note that the neuroendocrine function of IL-1 may be evolutionary conserved from invertebrates. In molluscs, CRF causes the production of biogenic amines as a stress response. This response is significantly reduced by IL-1 (175). Thus, this non-immunological IL-1 activity may have an ancient origin.

The non-immunological activities of microglia have been reviewed extensively. The readers are referred to these excellent reviews (11, 36, 176–178). Briefly, emerging evidences show microglia perform surveillance function during “resting state,” prune excessive synapse during development, contribute to adult neurogenesis, support neuronal survival, and modulate neurotransmission. Current research using advanced techniques in molecular biology, imaging and immunology has also identified significant heterogeneity in brain microglia in terms of morphology, gene expression profile, and cellular origin and fate (179). Some characteristics of subsets of microglia appear to be tightly linked with the potential neural function of these cells. For example, microglia from neurogenic regions are capable of substantial proliferation whereas microglia from non-neurogenic regions are not (180). Analysis of microglial expression patterns suggests that microglia from cerebellum and hippocampus appear immunologically more vigilant than microglia from other brain regions. Within Basal ganglia, microglia were found to show regional specific morphology, cell number, expression profile and activity relevant to motor activity and motion control, shaped by local cues (181). These findings demonstrate non-immunological functions of the microglia could be influenced by the specific neural circuitry they modulate. From this perspective, it is interesting to note that chronic unpredictable stress causes depression in association with a reduction of microglia numbers in hippocampus and stimulation of microglial activation by LPS or M-CSF restored microglia numbers and ameliorated stress-induced depression. In another study, chronic unpredictable stress was found to activate microglial cells in association with elevated CSF-1 expression in the prefrontal cortex (PFC), increase microglial phagocytosis of neuronal elements, and reduce dendritic spine density. Viral vector mediated knockdown of CSF-1 in the PFC blocked these effects and stress-induced anxiety- and depressive-like behavior (182). In neurodegenerative disease models, microglial production of proinflammatory cytokines and growth factors has been found to mediate neuroprotection against excitotoxicity (183, 184). In addition, microglia-mediated synaptic stripping was found to be neuroprotective following acute neural injury (185, 186). On the other hand, abnormal synaptic pruning have been observed in obsessive-compulsive disorder (OCD), indicating this mechanism might be pathogenic in OCD (187). These findings show non-immunological activities of microglia can be either neuroprotective or pathogenic depending on the specific circumstances.

## The Big Picture

The dizzying progress made in the field of CNS IL-1 and microglia has produced great excitement and confusion. It is clear CNS IL-1 and microglia have both immunological and non-immunological functions. These two types of functions may be separated not only by the physical barrier, such as the blood brain barrier, but also by an invisible barrier: the activation threshold of inflammatory cytokines. For example, IL-1 is able to activate neurons at 1,000-fold lower concentration than that is required for the activation of non-neuronal cells (188). It is possible that low levels of IL-1 acts in the CNS to perform non-immunological functions including non-immunological activation of microglia, which are involved in the remodeling of the CNS tissue. Higher concentration of IL-1 could engage non-neuronal cells of the CNS to produce neuroinflammation. Interestingly, although microglia is the main source of IL-1 production in the brain without infiltrated leukocytes, IL-1 does not directly stimulate microglial cell to produce IL-1 (189). Our unpublished results show IL-1 receptor is not expressed on resting microglia and CNS IL-1 induce microglia to produce IL-1 indirectly *via* cells of the blood-brain barrier and cells of CSF-brain barrier. The separation of the immunological and non-immunological functions of CNS IL-1 and microglia may be compromised during neural injury or aberrant neural activity. Thus the integrated perspective suggests that the disruption of the proper separation and coordination of the immunological and the non-immunological functions of CNS IL-1 and microglia might be a new way to think about the pathogenic potential of these two critical factors in CNS diseases.

Another important insight is that the detrimental effects of IL-1 and microglial activation does not always stem from immunological functions of these factors. A series studies from Centonze’s group showed that IL-1 and TNFα can cause hyper-excitation in neurons, causing excitotoxicity in MS (190). In addition, they found IL-1 could cause anxiety by blocking neuronal cannabinoid receptor 1-mediated control of GABAergic synapses (49, 100, 191, 192). Thus, aberrant non-immunological function of IL-1 can also contribute to disease progression.

**Figure 1:**
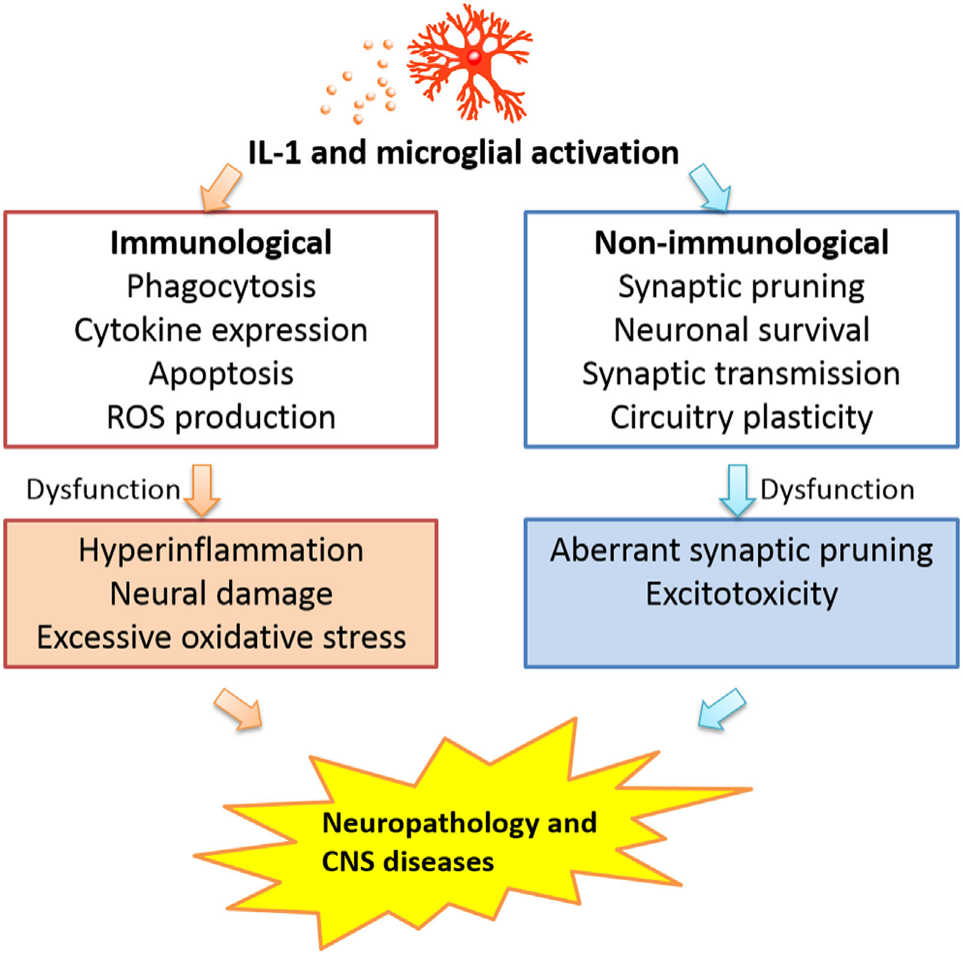
Interleukin-1 activity and microglial activation may influence CNS pathogenesis *via* dysregulation of either the immunological or the non-immunological functions of these factors.

The complex contribution of CNS IL-1 and microglia argues against a one size-fits-all approach to target these factors in treatment without careful considerations for the different phases of pathological processes. For acute brain tissue injury, blocking IL-1 activity and microglial activation at the early phase of the disease could be beneficial as this might dampen excessive neuroinflammation (15, 193); however, blocking later expression of low levels of IL-1 related to its promotion of clearing of debris and wound healing (194) may not be advisable. In chronic degenerative diseases, blockade of CNS IL-1 activity and microglial activation may also need to be titrated, such that the excessive activation of these factors may be attenuated, but the physiological neuroregulatory functions can be preserved. In an extreme case of an animal model of major depression, loss of microglia in hippocampus has been found to be a cause and stimulation of microglia proliferation can effectively alleviate behavioral symptoms of depression (195). Thus, one cannot assume that microglial activation, not deficiency, is always the cause of CNS diseases. The integrated perspective of microglial activation and IL-1 activity in the brain in regards to the pathogenesis of CNS diseases is presented in Figure 1 in which the immunological and non-immunological functions of IL-1 and microglial activation are seen as an integrated whole and dysregulation of either types of functions alone or in combination may contribute to disease progression.

## Author Contributions

NQ and XL cowrote this review.

## Conflict of Interest Statement

The authors declare that the research was conducted in the absence of any commercial or financial relationships that could be construed as a potential conflict of interest.
